# Effectiveness of mid-regional pro-adrenomedullin, compared to other biomarkers (including lymphocyte subpopulations and immunoglobulins), as a prognostic biomarker in COVID-19 critically ill patients: New evidence from a 15-month observational prospective study

**DOI:** 10.3389/fmed.2023.1122367

**Published:** 2023-03-24

**Authors:** Giorgia Montrucchio, Gabriele Sales, Eleonora Balzani, Davide Lombardo, Alice Giaccone, Giulia Cantù, Giulia D'Antonio, Francesca Rumbolo, Silvia Corcione, Umberto Simonetti, Chiara Bonetto, Marinella Zanierato, Vito Fanelli, Claudia Filippini, Giulio Mengozzi, Luca Brazzi

**Affiliations:** ^1^Department of Surgical Sciences, University of Turin, Turin, Italy; ^2^Department of Anesthesia, Critical Care and Emergency, “Città della Salute e della Scienza” Hospital, Turin, Italy; ^3^Clinical Biochemistry Laboratory, Department of Laboratory Medicine, “Città della Salute e della Scienza” Hospital, Turin, Italy; ^4^Department of Medical Sciences, University of Turin, Turin, Italy

**Keywords:** adrenomedullin, MR-proADM, biomarkers, COVID-19, SARS-CoV-2, intensive care, lymphocyte subpopulations, immunoglobulins

## Abstract

**Background:**

Mid-regional pro-adrenomedullin (MR-proADM), an endothelium-related peptide, is a predictor of death and multi-organ failure in respiratory infections and sepsis and seems to be effective in identifying COVID-19 severe forms. The study aims to evaluate the effectiveness of MR-proADM in comparison to routine inflammatory biomarkers, lymphocyte subpopulations, and immunoglobulin (Ig) at an intensive care unit (ICU) admission and over time in predicting mortality in patients with severe COVID-19.

**Methods:**

All adult patients with COVID-19 pneumonia admitted between March 2020 and June 2021 in the ICUs of a university hospital in Italy were enrolled. MR-proADM, lymphocyte subpopulations, Ig, and routine laboratory tests were measured within 48 h and on days 3 and 7. The log-rank test was used to compare survival curves with MR-proADM cutoff value of >1.5 nmol/L. Predictive ability was compared using the area under the curve (AUC) and 95% confidence interval (CI) of different receiver-operating characteristic curves.

**Results:**

A total of 209 patients, with high clinical severity [SOFA 7, IQR 4–9; SAPS II 52, IQR 41–59; median viral pneumonia mortality score (MuLBSTA)−11, IQR 9–13] were enrolled. ICU and overall mortality were 55.5 and 60.8%, respectively. Procalcitonin, lactate dehydrogenase, D-dimer, the N-terminal prohormone of brain natriuretic peptide, myoglobin, troponin, neutrophil count, lymphocyte count, and natural killer lymphocyte count were significantly different between survivors and non-survivors, while lymphocyte subpopulations and Ig were not different in the two groups. MR-proADM was significantly higher in non-survivors (1.17 ± 0.73 vs. 2.31 ± 2.63, *p* < 0.0001). A value of >1.5 nmol/L was an independent risk factor for mortality at day 28 [odds ratio of 1.9 (95% CI: 1.220–3.060)] after adjusting for age, lactate at admission, SOFA, MuLBSTA, superinfections, cardiovascular disease, and respiratory disease. On days 3 and 7 of the ICU stay, the MR-proADM trend evaluated within 48 h of admission maintained a correlation with mortality (*p* < 0.0001). Compared to all other biomarkers considered, the MR-proADM value within 48 h had the best accuracy in predicting mortality at day 28 [AUC = 0.695 (95% CI: 0.624–0.759)].

**Conclusion:**

MR-proADM seems to be the best biomarker for the stratification of mortality risk in critically ill patients with COVID-19. The Ig levels and lymphocyte subpopulations (except for natural killers) seem not to be correlated with mortality. Larger, multicentric studies are needed to confirm these findings.

## Introduction

Multiple indicators and biomarkers have been proposed, alone or in combination, to identify the most serious COVID-19 cases, but none proved to be entirely effective (1–3).

Pro-adrenomedullin is a multipotent regulatory peptide expressed in different tissues and organs and upregulated by inflammation, hypoxia, bacterial products, and shear stress. Mid-regional pro-ADM (MR-proADM), its stable precursor, is currently considered an effective biomarker of endothelial damage as its increase in plasma seems to correlate with disease severity (4). In fact, it plays a role in vascular permeability, inflammatory cascade, endothelial barrier regulation, and microcirculation performance, as well as essential in maintaining endothelial stability. The increase of MR-proADM has been demonstrated as an indicator of organ dysfunction and failure, and its predictive value has been highlighted in the context of respiratory infections, sepsis, and septic shock (5, 6).

Regarding COVID-19-related severe acute respiratory syndrome (SARS-CoV2), an association between MR-proADM levels and virus-induced endothelial damage is assumed, as endotheliitis has emerged as a prominent feature of the severe COVID-19 disease (7). MR-proADM levels seem to reflect disease progression, allowing the identification of patients who are at the most risk of developing a more severe form of the disease (8). It could even be able to predict SARS-CoV2-induced mortality, although the pathological mechanism underlying this correlation has not been fully clarified. In fact, most studies had limited dimensions and were designed in the context of a pandemic emergency, with heterogenicity of objectives and contexts (2, 9).

In an intensive care unit (ICU) setting, the evidence seems to be particularly limited. A recent systematic review and meta-analysis designed to clarify the use of MR-proADM in severe COVID-19 disease included 21 studies, published between 2020 and 2022 from European countries, addressing the use of pro-adrenomedullin in COVID-19 (9). The analysis included data from 252 patients, only in the ICU setting. At ICU admission, the average MR-proADM level was 1.01 vs. 1.64 in surviving (*n* = 182) and non-surviving (*n* = 70) patients, respectively, with the mean differences of MR-proADM values in survivors vs. non-survivors being −0.96 (95% CI: −1.26 to −0.65). Although MR-proADM levels at admission seem to predict mortality in the critical COVID-19 population, a cutoff value able to provide adequate guidance for the use of MR-proADM as an adequate prognostic index is still missing.

Moreover, there are no prospective observational studies exploring the relationship between the host immune response status of SARS-CoV-2-infected patients, both antibody and cellular immunity, and outcomes. It is known that the decreases in the number and function of some lymphocyte populations suggest close monitoring of patient immunological status, as lymphopenia, which is inversely proportional to the severity of the disease, is often reported in severe COVID-19 cases, while little is known about immunoglobulin changes and specific subtypes (2, 10, 11). To date, few studies have comprehensively assessed the dynamic changes in antibody levels and lymphocyte subpopulations in patients with COVID-19, and the results obtained so far are inconsistent and scarcely conclusive (12–14).

A previous preliminary analysis conducted on 57 patients by this group showed that MR-proADM values are higher in non-surviving ICU-COVID-19 patients, its predictive ability compared with other inflammatory biomarkers, and how its changes over time tend to be different in surviving and non-surviving patients (15). In this study, we analyze the evidence obtained by extending the data collection and adding the evaluation of the possible correlation between lymphocyte subpopulations and immunoglobulins.

## Methods

### Study design and population

It is an observational, prospective cohort study conducted in the regional referral ICU for the treatment of severe respiratory failure and extracorporeal membrane oxygenation (ECMO) support and in two temporary ICUs created to face the COVID-19 pandemic at the “Città della Salute e della Scienza” university hospital in Turin (Italy) in the period March 2020–June 2021.

The study was conducted according to the guidelines of the Declaration of Helsinki. Data acquisition and analysis were performed anonymously according to the protocol approved by the local Ethics Committee (number 0121515). Written informed consent was obtained in all compatible cases, in accordance with the local Ethics Committee's Italian regulation.

All consecutive adult patients requiring ICU admission and suffering from pneumonia caused by severe acute respiratory syndrome coronavirus 2 (SARS-CoV-2), confirmed by the real-time polymerase chain reaction (RT-PCR) on at least one respiratory tract specimen, were enrolled (16). All patients were treated according to current protocols for the management of patients with severe respiratory insufficiency in combination with the more updated directions emerging from the recent literature about COVID-19 pneumonia (17–21). All patients were followed up until they were discharged from the hospital to compute ICU, 28-day, and overall mortality, as well as the length of ICU and hospital stay.

Further information on the study protocol is reported in a previous article reporting on patients enrolled in the period March–June 2020 (15).

### Patients' data collection

The data collected from medical records included patients' demographic information, comorbidities, severity scores, clinical history, compliance with the respiratory system at ICU admission, days from onset of symptoms to ICU admission, days from hospital to ICU admission, adoption of rescue therapies (prone position, extracorporeal membrane oxygenation (ECMO), and inhaled nitric oxide), length of mechanical ventilation, and ECMO support.

The diagnosis of infections, including, bloodstream infection (BSI), ventilator-associated pneumonia (VAP), and etiologic pathogens, was made according to the European Center for Disease Prevention and Control's (ECDC) current definitions (19). Sepsis and septic shock were defined according to international guidelines (22, 23). Infections occurring in the first 48 h after ICU admission were considered co-infections, while infections occurring after 48 h or more were defined as super-infections (24).

Any administration of steroids and tocilizumab for COVID-19 (steroid treatment with intravenous methylprednisolone at any dosage and/or intravenous tocilizumab at 8 mg/kg repeated once) was recorded.

### Patients' laboratory data and imaging

All patients underwent the assessment of routine laboratory clinical tests, inflammatory and fibrinolysis biomarkers (C-reactive protein, CPR; procalcitonin, PCT; D-dimer; lactate dehydrogenase, LDH; and N-terminal prohormone brain natriuretic peptide, NT-pro-BNP). In addition, leukocyte, lymphocyte, and subpopulation {CD45+, CD3+, CD3+CD4+ [Th cells], CD3+CD8+, CD4+/CD8+, CD19+ (B lymphocytes), and CD16+CD56+ [NK cells]} counts were analyzed. All data and MR-proADM were collected within 48 h of ICU admission and on days 3 and 7 (see below).

Lymphocyte immunophenotyping was performed by an AQUIOS CL Flow Cytometry System using two separate combinations of four or five murine monoclonal antibody panels, each conjugated to a specific fluorochrome and specific for a different cell surface antigen (Kits Tetra-Panels 1 and 2), as per the manufacturer's instructions (Beckman Coulter, Inc., Brea, CA, USA).

Microbiological cultures of blood, bronchial aspirate, or bronchoalveolar samples, as well as radiologic investigation, such as chest X-rays or CT scans, were performed based on the intensivist's judgment in order to assess the progression of the disease.

### MR-proADM analysis

Samples of blood from an EDTA-containing tube were centrifuged at 4,000 rpm for 5 min, and then a plasma aliquot was immediately frozen and stored at −80°C. MR-proADM measures were determined using the B.R.A.H.M.S. KRYPTOR compact PLUS (Thermo Fisher Scientific, Hennigsdorf, Germany) automated method using the Time-Resolved Amplified Cryptate Emission (TRACE) technique. The detection limit of the assay was 0.05 nmol/L, while intra- and inter-assay coefficients of variation were under 4 and 11%, respectively.

### Statistical analyses

Summary data were presented as means and standard deviations or medians and interquartile ranges for continuous variables and as percentages for categorical variables. In univariate analysis, continuous variables were compared using the unpaired *t*-test or Wilcoxon-Mann-Whitney according to distribution type. Categorical variables were compared using the Fisher exact test or the Chi-square test, as appropriate.

For survival analysis, we used the Kaplan–Meier method, considering a period of 28 days. A log-rank test was used to assess the differences between survival curves considering the MR-proADM cutoff value of 1.5 nmol/L according to available studies on MR-proADM in patients with severe COVID-19 admitted to ICUs (9).

The effect of potential confounding factors was tested by a logistic regression model adjusted for age, lactate, SAPS II and SOFA score (23, 25), MuLBSTA score (26), the presence of superinfections, cardiovascular disease, and chronic lung disease, and the results are presented as odds ratios (OR) and 95% confidence intervals (CI).

The time course of the biomarker profiles in the different patient groups was tested using a generalized linear model for repeated measures.

The predictive ability of MR-proADM, PCT, D-dimer, LDH, and lymphocytes to discriminate surviving patients was compared using the area under the curve (AUC) and the 95% confidence interval (CI) of different receiver-operating characteristic curves (ROC) using the DeLong test.

All tests were two-sided, and the statistical significance level was set at 0.05. All analyses were performed with the R (3.5.0) and SAS software, version 9.4 (SAS Institute Inc., Cary, NC).

### Study outcomes

The 28-day all-cause mortality following ICU admission was the primary outcome. Patients were followed up from admission until hospital discharge or death. The length of stay (LoS) in the ICU and hospital were analyzed.

## Results

### Patients' characteristics

A total of 209 patients were enrolled in the period March 2020–June 2021 (Table 1).

**Table 1:** Patient clinical characteristics and outcomes.

**Clinical characteristics**	**Overall**	**Survivors**	**Non-survivors**	***P*-value**
*N* (%)	209	82 (39.2%)	127 (60.8%)	
Age, yrs	63.2 (10.9)	60.6 (11.1)	64.8 (10.6)	**0.0081**
Gender, male	159 (76.1)	60 (73.2)	99 (78.0)	0.5069
BMI	27.8 (25.4–31.3)	28.0 (25.0–31.3)	27.7 (25.4–31.3)	0.82
Lactate	3.86 (±18.9)	1.65 (±1.8)	5.19 (±23.8)	**<0.0001**
SOFA on admission	7 (4–9)	5 (4–7)	8 (5–10)	**<0.0001**
MuLBSTA on admission	11 (9–13)	10 (7–13)	13 (9–15)	**<0.0001**
SAPS II (*N* = 152)	52 (41–59)	44.5 (39–53)	54 (46–61)	**0.0002**
Patient transferred from other ICUs	88 (42)	30 (36.6)	58 (45.7)	0.2008
Comorbidities ≥ 3	97 (46.4)	34 (41.5)	63 (49.6)	0.2491
Arterial hypertension	132 (63.2)	47 (57.3)	85 (66.9)	0.1596
Cardiovascular disease	36 (17.2)	7 (8.5)	29 (22.8)	**0.0083**
Chronic lung disease	28 (13.4)	5 (6.1)	23 (18.1)	**0.0128**
Chronic renal failure	14 (6.7)	4 (4.9)	10 (7.9)	0.3976
Neurologic disease	12 (12.77)	4 (11.43)	8 (13.56)	1.0
Neoplasm, solid	5 (2.40)	0	5 (2.40)	0.1591
Neoplasm, hematologic	8 (3.86)	2 (0.97)	6 (2.90)	0.4826
Autoimmune disorder	20 (9.6)	10 (12.2)	10 (7.9)	0.3399
Immunosuppressive therapy	14 (6.7)	7 (8.5)	7 (5.5)	0.4087
Diabetes mellitus	46 (22)	13 (15.9)	33 (26)	0.0904
**Ventilation characteristics**				
Patient underwent CPAP	143 (69.1)	50 (62.5)	93 (73.3)	0.1230
Patient underwent NIV	153 (73.9)	56 (69.1)	97 (77)	0.2564
Invasive mechanical ventilation at arrival	133 (64.3)	49 (59.8)	84 (67.2)	0.4194
Non-invasive mechanical ventilation at arrival	74 (37.8%)	33 (40.2%)	41 (32.8%)	0.2744
Invasive mechanical ventilation during ICU stay	187 (89.9)	65 (80.3)	122 (96.1)	**0.0002**
Non-invasive mechanical ventilation only	21 (10.1%)	16 (19.6%)	5 (3.9%)	
Invasive mechanical ventilation days [*N* = 192]	12 (5–20)	8 (4–13)	14 (7–22)	**0.0007**
**Treatments**				
Steroids	152 (74.9)	53 (68)	99 (79.2)	0.0957
Tocilizumab	44 (21.1)	17 (20.7)	27 (21.3)	1
Curarization	174 (87.9)	57 (75)	117 (95.9)	**<0.0001**
Prone positioning	154 (73.7)	54 (65.9)	100 (78.7)	0.0530
Inhalator nitric oxid	39 (18.8)	4 (4.9)	35 (27.6)	**<0.001**
Acute kidney injury	36 (17.2)	8 (9.8)	28 (22.1)	**0.0244**
RRT during our ICU stay	21 (10.1)	1 (1.2)	20 (15.8)	**0.0003**
Vasopressors during our ICU stay	142 (71.7)	34 (44.7)	108 (88.5)	**<0.0001**
ECMO at arrival	40 (19.1)	8 (9.8)	32 (25.2)	**0.0065**
ECMO during ICU stay	47 (22.5)	8 (9.8)	39 (30.7)	**0.0003**
RRT, total days of [*N* = 21]	6 (2–9)	1 (14)	4.5 (2–8.5)	**0.0003**
ECMO, total days of [*N* = 47]	16 (9–23)	14 (6.5–21)	16 (9–23)	0.5145
**Bacterial infections**				
Co-infections within 48 h	27 (12.9)	4 (4.9)	23 (18.1)	**0.0055**
Super-infections during ICU stay	134 (64.1)	34 (41.5)	100 (78.7)	**<0.0001**
Septic shock during ICU stay	61 (29.2)	6 (7.3)	55 (43.3)	**<0.0001**
**Outcomes**				
28 days mortality	107 (51.2)			
Overall mortality	127 (60.8)			
ICU mortality	116 (55.5)			
ICU LOS	13 (8–22)	10 (6–19)	16 (10–23)	**0.0042**
Hospital LOS	23 (15–32)	28 (16–38)	22 (15–30)	0.0657

ICU, intensive care unit; BMI, body mass index; ECMO, extracorporeal membrane oxygenation; SAPS, simplified acute physiology score; SOFA, sequential organ failure assessment; RRT, renal replacement therapy; LOS, length of stay. The bold values indicate statistical significance.

The SOFA and SAPS II (23, 25) scores at ICU admission were 7 (IQR 4–9) and 52 (IQR 41–59), respectively, with a significantly lower median value in survivors [5 (IQR 4–7) vs. 8 (IQR 5–10)] and [44.5 (IQR 39–53) vs. 54 (IQR 46–61), respectively] (*p* < 0.0001 and 0.0002, respectively).

The median viral pneumonia mortality score (MuLBSTA) (26) and mean lactate value at admission were 11 (IQR 9–13) and 3.86 (SD 18.9), respectively, with significantly lower values in survivors [10 (IQR 7–13) vs. 13 (IQR 9–15)] and 1.65 mmol/L vs. 5.19 mmol/L, respectively (a *p* < 0.0001 for both comparisons).

A total of 133 (64.3%) patients were treated with mechanical ventilation on ICU admission and 187 (89.9%) during hospitalization. Notably, 21 (10.1%) patients required only non-invasive ventilatory support. Mortality in the ventilatory support group (invasive or non-invasive) was significantly higher (*p*-value 0.0002). The cohort of deceased patients was then characterized by a longer mechanical ventilation period of 14 (IQR 7–22), 12 (IQR 5–20), and 8 (IQR 4–13) (*p*-value 0.0007) and by increased use of neuromuscular blockers, nitric oxide, and vasopressors (*p*-value < 0.0001, < 0.001, and < 0.0001, respectively).

A total of 36 patients (17.2%) had an acute renal failure during ICU admission (both acute and developed prior to ICU admission), and 21 patients (10.1%) received renal replacement therapy (RRT) for a median time of 6 days (IQR 2–9). The cohort of surviving patients showed a lower frequency of renal failure (*p* = 0.0244) and a shorter duration of RRT (14 vs. 4.5 days, IQR 2–8.5) (*p* = 0.0003).

According to the current literature (24), co-infection is defined as infections that occurred in the first 48 h of admission, and in this study, it occurred in 20 (9.6%) patients within the first 24 h and 27 (12.9%) during the first 48 h of ICU stay. Overall, during the whole ICU admission, 134 (64.1%) patients contracted at least one superinfection. Superinfections at any time were lower in survivors (*p* < 0.0280, <0.0055, and <0.0001, respectively). The septic shock occurred in 61 (29.2%) superinfected patients, with a statistically significant difference between survivors and non-survivors (*p* < 0.0001).

Overall mortality was 60.8%, while ICU and 28-day mortality were 55.5 and 51.2%, respectively. The median ICU and hospital length of stay were 13 and 23 days, respectively. Mortality was statistically correlated with age (*p* = 0.0081), cardiovascular, and pulmonary comorbidities (*p* = 0.0083 and 0.0128, respectively).

### Laboratory tests/biomarkers

**Figure 1:**
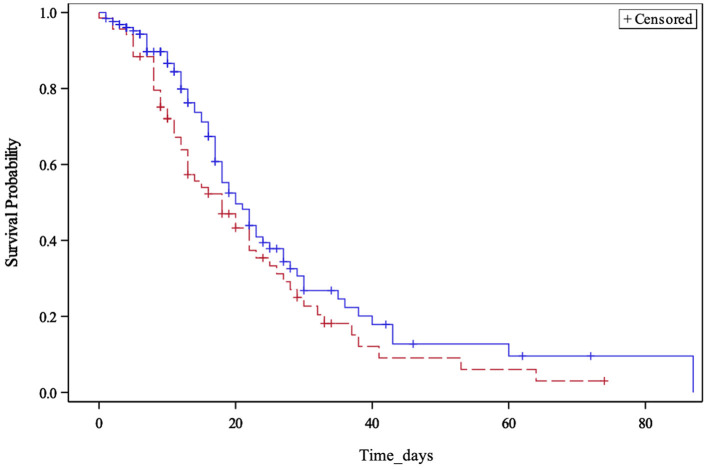
Kaplan–Meier survival curve. Stratification of patients with mid-regional pro-adrenomedullin (MR-proADM) levels greater or less than 1.5 nmol/L at an intensive care unit admission.

**Figure 2:**
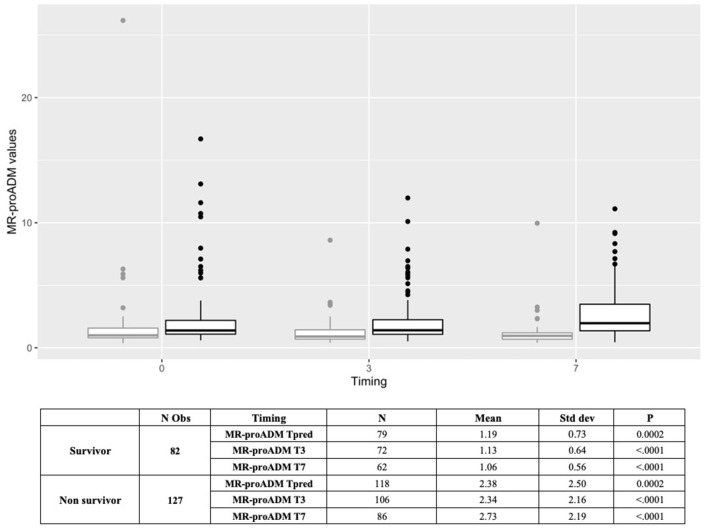
Boxplot representing the trend of MR-proADM over time in overall population (*N* = 209) (Tpred, predictive value; T0, first available value in 48 h; T3, value at day 3; T7, value at day 7). Outcome: survivors (gray); non-survivors (black). MR-proADM, mid-regional pro-adrenomedullin; Tpred (predictive value), first available value in 48 h; *N*, number.

D-dimer, LDH, NT-proBNP, PCT, myoglobin, troponin-I (hs), neutrophils, lymphocytes, natural killer lymphocytes, and interleukin-6, measured within the first 48 h, were statistically different between survivors and non-survivors (Table 2). Similarly, MR-proADM values, measured within the first 48 h, were significantly higher in non-survivors (1.17 ± 0.73 vs. 2.31 ± 2.63, *p* < 0.0001). Patients with a predictive MR-proADM value exceeding the cutoff value of 1.5 nmol/L had higher mortality (*p* = 0.001) (Figure 1). Even the trend over time of MR-proADM was significantly different between survivors and non-survivors (Figure 2).

**Table 2:** Values of laboratory parameters and biomarkers (first available measures performed within 48 h from ICU admission).

**Predictive values**	**Overall (*N* = 209)**	**Survivors (*N* = 82)**	**Non-survivors (*N* = 127)**	***P*-value**
MR-proADM, *nmol/L, mean ± std* [*N* = 198]	1.85 (±2.16)	1.17 (±0.73)	2.31 (±2.63)	**<0.0001**
D-dimer, *ng/mL* [*N* = 205]	9,235.7 (±19,150)	7,143.7 (±17,342.1)	10,574.6 (±20,176.2)	**0.0019**
LDH, *UI/L* [*N* = 205]	942.2 (±544.4)	780.3 (±281.3)	1,048.1 (±641.5)	**<0.0001**
NT-proBNP*, ng/L [N = 197]*	2,049.1 (±7,056.1)	669.5 (±1,215.7)	2,915.6 (±8,856.3)	**<0.0001**
C-RP, *mg/L* [*N* = 208]	115.2 (±98.1)	109.3 (±99.4)	118.9 (±97.5)	0.3562
PCT, *μg/L* [*N* = 207]	2.30 (±6.81)	0.86 (±2.16)	3.20 (±8.44)	**0.0251**
Myoglobin, *μg/L* [*N* = 193]	186.3 (±307.1)	156.9 (±290)	205 (±317.3)	**0.0356**
CK, *UI/L* [*N* = 206]	254.5 (±592.6)	182.8 (±235.9)	301 (±734.3)	0.7823
Copeptin, *pmol/L* [*N* = 154]	34.0 (±50)	30.3 (±40.6)	36.3 (±55.3)	0.7712
Ferritin *ng/ml, mean ± std* [*N* = 182]	1,931.3 (±2,367.9)	1,610.3 (±1,414.7)	2,141.4 (±2,809.7)	0.1620
Troponin-I hs, *ng/L* [*N* = 193]	43.3 (±112.9)	24.4 (±57.3)	55.1 (135.5)	**<0.0001**
Neutrophils, cell x 10*9/L [*N* = 206]	11 (±7.9)	9.65 (±9.1)	11.9 (±6.9)	**0.0012**
Lymphocytes, cell x 10*9/L [*N* = 206]	0.95 (±2.75)	0.87 (±1.0)	0.99 (±3.45)	**0.0157**
Lymphocytes B (CD19+) [*N* = 157]	155.8 (±201.2)	160.7 (±241.7)	152.6 (±171)	0.3044
Lymphocytes T (CD3+) [*N* = 157]	388.4 (±256.7)	407.1 (±227.8)	376.2 (±274.4)	0.1179
Lymphocytes T helper (CD3+CD4+) [*N* = 157]	271.9 (±200.5)	282 (±181)	265.4 (±212.9)	0.1781
Lymphocytes T suppressor (CD3+CD8+) [*N* = 157]	107.6 (±73.9)	111.4 (±69.4)	105.2 (±76.9)	0.2985
Lymphocytes natural killer (CD16+CD56+) [*N* = 157]	61.9 (±60)	76.5 (±71.5)	52.3 (±49.3)	**0.0078**
Immunoglobulin A, *g/L* [*N* = 164]	262.6 (±139.3)	254 (±111.7)	267.5 (±153.3)	0.7808
Immunoglobulin G, *g/L* [*N* = 163]	922 (±475.4)	873.4 (±200.1)	949.5 (±575.2)	0.8332
Immunoglobulin M, *g/L* [*N* = 163]	113.8 (±126)	110.5 (±85.5)	115.6 (±144.4)	0.8103
Interleukin 6, *pg/mL* [*N* = 109]	463.9 (±1,074.1)	343.6 (±1,635.7)	528.3 (±1,747.7)	**0.0093**

MR-proADM, mid-regional pro-adrenomedullin; LDH, lactate dehydrogenase; NT-proBNP, N-terminal prohormone of brain natriuretic peptide; CRP, C-reactive protein; PCT, procalcitonin; CK, creatine kinase. The bold values indicate statistical significance.

**Figure 3:**
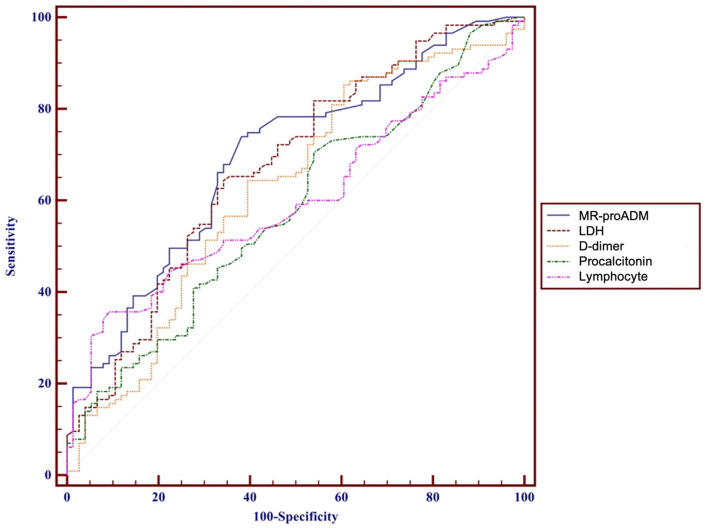
ROC curves performance of MR-proADM (blue), LDH (brown), D-dimer (yellow), PCT (green), Ly (pink) predictive values, and their comparison for predicting 28-day mortality. MR-proADM, mid-regional pro-adrenomedullin; LDH, lactate dehydrogenase; PCT, procalcitonin; Ly, lymphocytes; AUC, area under the curve.

Overall, MR-proADM was found to have the best predictive ability compared to other biomarkers (area under the curve, AUC: 0.695 [95% CI: 0.624–0.759]; LDH, AUC = 0.674 [95% CI: 0.603–0.740]; PCT, AUC = 0.581 [95% CI: 0.508–0.652]; D-dimer, AUC = 0.626 [95% CI: 0.553–0.695]; lymphocytes count, AUC = 0.598 [95% CI: 0.525–0.668]) (Figure 3). Even the combination of different biomarkers was unable to produce a better result.

These results were confirmed by the logistic regression model adjusted for age, lactate, SOFA and MuLBSTA scores (evaluated within 48 h of ICU admission), the presence of superinfections, cardiovascular disease, and chronic pulmonary disease, which confirmed a statistically significant odds ratio equal to 1.9 [95% CI: 1.220–3.060] for MR-proADM values higher than 1.5 nmol/L (Table 3).

**Table 3:** Multivariate logistic regression analysis for mortality.

**Effect**	**OR**	**95% CI**
Predictive MR-proADM	**1.932**	1.220–3.060
SOFA	**1.221**	1.052–1.416
MuLBSTA	**1.244**	1.089–1.420
Lactate	1.155	0.896–1.489
Superinfections	**8.862**	3.532–22.233
Cardiovascular disease	3.400	0.915–12.632
COPD	**5.673**	1.266–25.429

CI, confidence interval; MR-proADM, mid-regional pro-adrenomedullin; SOFA, sequential organ failure assessment; MuLBSTA, multilobular infiltration, hypo-lymphocytosis, bacterial coinfection, smoking history, hypertension, and age; COPD, chronic obstructive pulmonary disease. The bold values indicate statistical significance.

**Figure 4:**
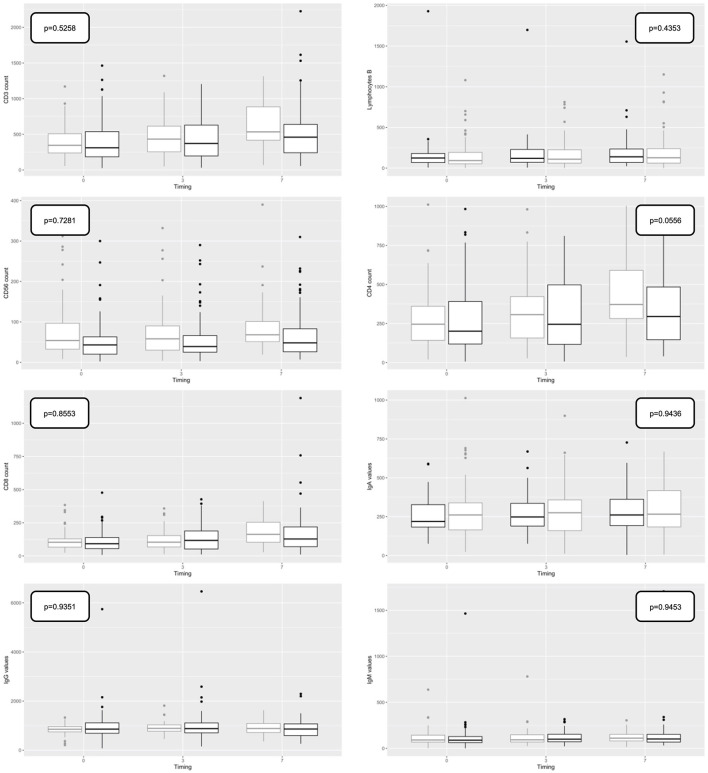
Boxplot representing the trend of Immunoglobulin (Ig) (subtypes IgM, IgG, and IgA) and lymphocytes (lymphocytes B, CD19, CD3, CD3/CD4, CD4/CD8, and CD16/CD56) predictive values over time in overall population (T0, first available value in 48 h; T3, value at day 3; T7, value at day 7). Outcome: survivors (gray); non-survivors (black).

Lymphocyte subpopulations, such as CD3, CD4, CD8, and immunoglobulin values (IgA, IgG, and IgM) were not statistically different between survivors and non-survivors within 48 h of admission or during the time (Figure 4).

### Patients with veno-venous ECMO support

A total of 47 patients (22.5%) undergoing veno-venous ECMO (vv-ECMO) support were enrolled (40 before ICU admission and 7 during ICU stay). Of them, 39 died, resulting in overall mortality of 83% in ECMO patients and statistically higher in comparison to the non-ECMO cohort (*p* = 0.0003).

**Figure 5:**
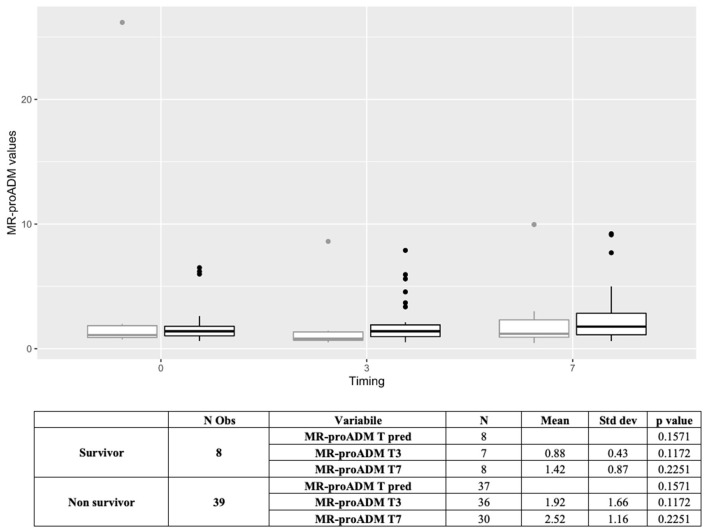
Boxplot representing the trend of MR-proADM predictive values over time in extracorporeal membrane oxygenation (ECMO) population (*N* = 47) (Tpred, predictive value; T0, first available value in 48 h; T3, value at day 3; T7, value at day 7). Outcome: survivors (gray); non-survivors (black). MR-proADM, mid-regional pro-adrenomedullin; *N*, number.

MR-proADM trend analysis in the subgroup of patients undergoing vv-ECMO failed to evidence statistically significant differences between surviving and non-surviving patients (*p* = 0.562), and MR-proADM values on days 3 and 7 suggest a difference between groups, although not statistically significant (*p* = 0.08). Considering other standard biomarkers, only the lymphocyte count was found significantly different between survivors and non-survivors (*p* = 0.0471) (Figure 5).

## Discussion

This prospective, observational study, on a cohort of over 200 critically ill ICU-COVID-19 patients, confirms the validity of the biomarker MR-proADM in predicting mortality (9, 15). Its values, measured within the first 48 h of ICU admission, proved to be significantly higher in patients with a fatal outcome and revealed an ability to discriminate between surviving and non-surviving patients better than other biomarkers commonly used in ICU, such as PCT, C-RP, LDH, and D-dimer. Moreover, patients who had a predictive value of MR-proADM >1.5 nmol/L showed higher mortality, confirming this value as a possible cutoff value in this population [OR of 1.9 (95% CI: 1.220–3.060)] (9).

The increase in MR-proADM, due to a dose-response mechanism induced by the host-pathogen interaction, seems to occur in the initial stage of pathogen recognition, i.e., at the time of hospital admission or even earlier. However, our findings confirm that the increase persists in the following days in accordance with the evolution of the disease, as shown by the trend of the values (48 h, day 3, and day 7). We consider this temporal trend analysis particularly interesting as it could help to overcome some limits of the single-value evaluation.

Other indicators, less studied in this patient setting, such as lymphocyte subpopulations or the level of serum immunoglobulins (subgroups IgA, IgM, and IgG), were not able to predict disease severity and mortality. This is in line with recent findings in the literature (13, 14), which seem to suggest that no defined prognostic value can be uniquely attributed to immunologic biomarkers or cytokines.

Our cohort is relatively young, with a predominance of the male gender (76.1%), without impact on mortality, unlike the results reported by the ISARIC clinical characterization group (27–29). Patients presented with at least one comorbidity in most cases (89%), and three or more in 46.4% of cases, in line with literature evidence (30). Among comorbidities, only cardiovascular diseases (*p* = 0.0083), already known risk factors (31, 32), and chronic pulmonary diseases (*p* = 0.0128) were demonstrated to play a significant role in the univariate analysis.

All clinical severity scores (SOFA, median value 7; MuLBSTA, median value 11; and SAPS II, median value 52) highlighted how our population has greater severity compared to previous studies (33–35). This is also confirmed by the high percentage of patients requiring invasive mechanical ventilation (89.9%), vasopressors (71.7%), renal replacement therapy (RRT) (10.1%), and developing septic shock (29.2%). As a result, the length of ICU and hospital stay (13 and 23 days, respectively), and ICU and hospital mortality rates (55.5 and 61%, respectively) were higher than those observed in other studies (36). In our population, co-infections and superinfections are relatively common complications of severe COVID-19; in particular, in our analysis, the presence of superinfections was associated with increased odds of death, in line with other studies showing a positive association between co-infection or superinfection and an increased risk of death, especially in ICU (24).

The biomarkers we analyzed in this study were those already in use or deemed potentially useful at the beginning of the pandemic and during subsequent waves. Many of these were elevated on admission (LDH, D-dimer, NT-proBNP, PCT, myoglobin, CK, ferritin, troponin-I (hs), and IL6). Among these, the mean values of LDH (*p* < 0.0001), D-dimer (*p*-value 0.0019), NT-proBNP (*p*-value < 0.0001), PCT (*p*-value 0.0251), myoglobin (*p*-value 0.0356), troponin-I hs (*p*-value < 0.0001), and IL-6 (*p*-value 0.0093) seem to be able to identify patients subsequently burdened by major mortality. C-RP, PCT, ferritin, IL-6, and LDH predictive values were found to be altered in most of our critical population, in line with previous literature (2).

The role of generic inflammation biomarkers, to which much attention was given at the beginning of the pandemic, has been greatly reduced in light of the most recent findings, as the elevation of inflammatory biomarkers as well as cytokine parameters does not seem to be able to provide a clear prognostic indication (14, 37–39). In our population, the analysis of the AUC for PCT, LDH, D-dimer, and lymphocytes suggested underperformance compared to MR-proADM (Figure 3) and no predictive ability for C-RP, ferritin, copeptin, lymphocytes subpopulation, and immunoglobulin (Table 2). MR-proADM, instead, appears to be a biomarker with a strong prognostic value, as supported by a series of experimental evidence attributing to ADM an important role in the regulation of vascular and endothelial barrier permeability, inflammatory mediators, and microcirculation (40, 41).

ROC curve analysis of MR-proADM showed that this biomarker has a significantly greater predictive capacity than other biomarkers. The increase in MR-proADM, resulting from a dose-response mechanism induced by the host-pathogen interaction, appears to occur in the early stages of pathogen recognition, at the time of hospital admission or even before. However, it is interesting to note that the increase persists in the following days in accordance with the evolution of the disease, underlying the additional value of this biomarker in predicting the patient's outcome. In line with the evidence of other recent studies, which also proposed the analysis of the trend over time of this marker in the course of sepsis (42), we highlighted how MR-proADM could play a role in monitoring the progression of the disease (8), as evidenced by the results obtained on days 3 and 7. This analysis appeared to be a peculiarity of great interest in this work since it could allow the overcoming of some limitations of the punctual evaluations. In fact, this new biological marker of endothelial damage and vascular permeability could have an important clinical impact, particularly in the ICU setting. As it has been reported that MR-proADM can contribute to the correct triage of patients with COVID-19 in the emergency department (6, 42, 43), its role in guiding new early diagnostic interventions and making possible the anticipation of more intensive treatment, regardless of the causative pathogen, such as bacteria, fungi, or viruses, could be crucial in the ICU setting. MR-proADM could also be used, together with the traditional severity score on admission (SAPS, APACHE, etc.), to predict the outcome but also to allow better monitoring of the patient's course, to evaluate the effectiveness of treatments, and to anticipate an early identification of any possible worsening, especially if collected repeatedly over time. Finally, the MR-proADM trend might safely guide transfer from ICU to general wards without increasing the number of re-admissions and/or mortality. Further studies are needed to confirm these hypotheses.

A further innovative aspect of our study is represented by the analysis of the so-called immunologic biomarkers, namely, lymphocyte subpopulations and circulating levels of immunoglobulins Ig (A, M, and G) (13, 14). It has in fact been hypothesized that the pulmonary involvement, typical of severe forms of COVID-19, may be due to a dysregulated systemic inflammatory response induced by a macrophage activation syndrome that mimics acquired hemophagocytic lymphocytic histiocytosis (43–45). This might be reflected in severe lymphopenia with an inflammatory response and release of a cytokine cascade, previously considered markers of disease severity (46, 47). In fact, it is known that T cells, including CD4+ and CD8+, have an important antiviral role in balancing the response against pathogens and the risk of developing autoimmunity or excessive inflammation. The reduction of CD8+ T cells and B cells and the consequent increase in the CD4/CD8 ratio has been reported in numerous cases of critically ill patients with COVID-19. Furthermore, the reduction in lymphocyte counts correlates with the severity of SARS-CoV-2 disease and represents an important risk factor for a poor prognosis (48, 49).

Lymphocyte subpopulations were previously evaluated by Zhang et al. in a study that found a statistically significant difference in terms of prognosis and in-hospital length of stay in patients with mild, severe, and critical COVID-19 disease (50). While those data confirmed a clear depletion of lymphocyte subpopulations in patients suffering from severe vs. mild disease, the study population was more heterogeneous than that included in ours. It is precisely the homogeneity of the population we enrolled that we believe might explain the absence of statistical significance observed in our study, similar to the results reported by Pan et al. previously (51). Indeed, the pattern of lymphocyte subpopulations in patients with COVID-19 has been described in conflicting literature that, however, focused more on the severity of the presentation of the disease than on mortality. Additionally, the possible impact of corticosteroids on lymphocyte numbers and subpopulations deserves a note. However, the duration of our study covered a rather large period, during which the indications in the literature with respect to steroid therapy changed in terms of indication, duration, and dosage. Due to this heterogeneity, it was not possible to conduct a focused analysis differentiated by steroid type and dosages.

Immunoglobulins M (IgM) are known to represent the first line of defense during viral infections, prior to the generation of the high-affinity immunoglobulin G (IgG) adaptive immune response, which is important for long-term immunity and immunological memory. The specific antibody response against SARS-CoV-2 is related to the severity of the disease and the prognosis of patients with COVID-19. In the previous phases of the pandemic, the impaired immunological response was hypothesized as a potential target for the treatment of the COVID-19 disease, so much so that treatment with intravenous immunoglobulin IgG (IVIG) was proposed with the aim of mitigating the immunosuppression caused by the virus and guaranteeing broad-spectrum immune protection, not without adverse events (52). Treatment with IVIG has neither been standardized in COVID-19 nor has the assessment of the pre-treatment IgG level (53).

As previously said, however, studies in the literature are inconclusive. A German study conducted on 62 patients found that patients with lower IgG levels are characterized by more severe forms of the disease, an earlier need for ICU admission, a lower P/F ratio, a higher SOFA, a higher incidence of AKI, and lower lymphocyte levels. In these patients, the clinical course is characterized by a higher mortality rate (46.2 vs. 14.3%; *p* = 0.012), a longer ICU stay [28 (6-48) vs. 12 (3-18) days; *p* = 0.012], and hospital length of stay [30 (22-50) vs. 18 (9-24) days (*p* = 0.004)] (54). Another study conducted on 707 patients looked at both subpopulations and IgM and IgG levels in patients with COVID-19, finding low lymphocyte levels in more severe patients and lower IgM and IgG levels in the most severe cases (55). Nevertheless, no statistically significant correlation was found between the total number of Ly T, CD4+, and CD8+ cells and those of Ig. It was then observed that the total number of T cells, CD4+, and CD8+ gradually recovered in critically ill patients who had a favorable course while remaining low in those with moderate forms. The production of IgM and IgG was delayed in the critically ill group.

In this situation of uncertainty on the real impact of immunoglobulins and lymphocyte subpopulations in the critically ill ICU patients context, we do believe that our data, which show no statistically significant correlation between IgG, IgM, and IgA levels and mortality, need to be confirmed in larger studies with a less severe comparison population and no potentially confounding factors (in particular, comorbidities and superinfections).

Finally, the analysis of the vv-ECMO cohort (47 patients, 22.5% of the total) certainly deserves a comment (56–58). In this subgroup, no differences in MR-proADM values were evidenced between survivors and non-survivors probably due to the confounding factor represented by the intrinsic endotheliitis linked to ECMO support and the high mortality observed in this cohort of patients (82.9%) (59). However, it should be noted that, even in this subgroup, the MR-proADM values measured on days 3 and 7 were higher in non-survivors without reaching statistical significance (*p* = 0.08). Further studies with a larger sample size are needed to better understand the variables that influence MR-proADM trends in the subpopulation of critically ill patients with COVID-19 undergoing vv-ECMO.

This study has limitations: (1) it is a monocentric experience (although in different ICUs); (2) it refers to a highly complex university center, receiving critical patients as secondary hospitalization and vv-ECMO support; (3) the high mortality of patients and their turn-over in a pandemic period may have created a selection bias. Furthermore, due to the pandemic context, there is a lack of a comparison population, represented by patients admitted to ordinary or semi-intensive hospital wards. Finally, the possible impact of confounding factors, such as bacterial superinfections, which are widely represented in our population (60–62), and cardiovascular and renal dysfunction cannot be clearly defined.

In the near future, the use of biomarkers, especially MR-proADM, would be focused to obtain possible early indications about specific treatment (e.g., pharmacological treatment) and patients/resource allocation (ICU vs. ordinary ward), including a comparison population with different severity and different stage of organ dysfunction (e.g., ARDS, hemodynamic disorders, and superinfections).

## Conclusion

MR-proADM can effectively predict the risk of death in severe COVID-19 patients. Higher MR-proADM values at ICU admission can identify patients with worse outcomes. In addition, measuring the temporal evolution of MR-proADM values with repeated monitoring could help in assessing clinical progression.

The values of IgA, IgM, and IgG, as well as lymphocyte subpopulations (except for natural killers), do not appear to be related to outcome, even if the peculiarities of the enrolled cohort may have influenced this analysis, which should be confirmed by larger studies that can better assess the role of possible confounding factors.

## Data availability statement

The raw data supporting the conclusions of this article will be made available by the authors, without undue reservation.

## Ethics statement

The studies involving human participants were reviewed and approved by Ethics Committee: Comitato Etico Interaziendale A.O.U. Città della Salute e della Scienza di Torino—A.O. Ordine Mauriziano—A.S.L. Città di Torino; ethics approval number 0121515. The patients/participants provided their written informed consent to participate in this study.

## Author contributions

GMo, GS, and LB: conceptualization. GMo, FR, GMe, and CF: methodology. GMo, EB, and CF: formal analysis. GMo, GS, EB, DL, AG, GC, GD'A, US, and CB: data curation. GMo and EB: writing—original draft preparation. GS, VF, US, CB, MZ, SC, and LB: writing—review and editing. LB and GMe: supervision. All authors have read and agreed to the published version of the manuscript.
